# A new index for characterizing micro-bead motion in a flow induced by ciliary beating: Part I, experimental analysis

**DOI:** 10.1371/journal.pcbi.1005605

**Published:** 2017-07-14

**Authors:** Mathieu Bottier, Sylvain Blanchon, Gabriel Pelle, Emilie Bequignon, Daniel Isabey, André Coste, Estelle Escudier, James B. Grotberg, Jean-François Papon, Marcel Filoche, Bruno Louis

**Affiliations:** 1 Inserm U955, Equipe 13, Créteil, France; 2 Université Paris-Est, Faculté de médecine, Créteil, France; 3 CNRS, ERL 7240, Créteil, France; 4 CHU Toulouse, Hôpital des Enfants, Service de pneumologie-allergologie pédiatrique, Toulouse, France; 5 AP-HP, Hôpital H. Mondor-A. Chenevier, Service d’ORL et de chirurgie cervico-faciale, Créteil, France; 6 Hôpital intercommunal, Service d’ORL et de chirurgie cervico-faciale, Créteil, France; 7 Inserm, U933, Paris, France; 8 Université Pierre et Marie Curie, U933, Paris, France; 9 AP-HP, Hôpital Armand-Trousseau, Service de génétique et d’embryologie médicale, Paris, France; 10 Department of Biomedical Engineering, University of Michigan, Ann Arbor, MI, USA; 11 AP-HP, Hôpital Bicêtre, Service d’ORL et de chirurgie cervico-faciale, Le Kremlin-Bicêtre, France; 12 Physique de la Matière Condensée, Ecole Polytechnique, CNRS, Université Paris-Saclay, Palaiseau, France; University of California Riverside, UNITED STATES

## Abstract

Mucociliary clearance is one of the major lines of defense of the respiratory system. The mucus layer coating the pulmonary airways is moved along and out of the lung by the activity of motile cilia, thus expelling the particles trapped in it. Here we compare *ex vivo* measurements of a Newtonian flow induced by cilia beating (using micro-beads as tracers) and a mathematical model of this fluid flow, presented in greater detail in a second companion article. Samples of nasal epithelial cells placed in water are recorded by high-speed video-microscopy and ciliary beat pattern is inferred. Automatic tracking of micro-beads, used as markers of the flow generated by cilia motion, enables us also to assess the velocity profile as a function of the distance above the cilia. This profile is shown to be essentially parabolic. The obtained experimental data are used to feed a 2D mathematical and numerical model of the coupling between cilia, fluid, and micro-bead motion. From the model and the experimental measurements, the shear stress exerted by the cilia is deduced. Finally, this shear stress, which can easily be measured in the clinical setting, is proposed as a new index for characterizing the efficiency of ciliary beating.

## Author summary

Mucociliary clearance is the first line of defense of the human pulmonary airways. Mucus transporting debris, particles, microorganisms and pollutants is carried away by the coordinated motion of cilia beating at the surface of the airway epithelium. We present here an experimental, mathematical and numerical study aiming at defining a global index for assessing the efficiency of this beating. We measure experimentally the ciliary beat frequency, ciliary beat amplitude, and metachronal wavelength on ciliated edges obtained from nasal brushing. Properties of fluid motion are simultaneously extracted from micro-bead tracking next to the ciliated edge. A mathematical and numerical model is developed to describe the fluid motion induced by the cilia tips considered as a moving wall. Experimental and numerical results show that the bead velocity is a parabolic function of the distance to the wall. It allows us to infer the shear stress exerted by the cilia on fluid from micro-bead tracking. This quantity is proposed as a universal index characterizing the beating efficiency, which can be extracted in the current clinical setting.

## Introduction

Mucociliary clearance is one of the major defense mechanisms of the respiratory airway system. The mucus layer coating the epithelial surface of the airways filters the inhaled air by trapping potentially harmful material (fungi, bacteria and other particles) [1–4]. This mucus layer is continuously carried away and out of the airways by the activity of motile cilia. Neighboring cilia beat in an organized manner with a small phase lag, their tips creating an undulating surface on top of the cilia layer which deforms in a wave-like fashion called the metachronal wave [5–7].

The beat pattern of an individual cilium displays a two-stroke effective-recovery motion [8]. During the effective stroke, cilia beat forwards and engage with the mucous layer, propelling it forward. In contrast, during the recovery stroke, they return to their initial position in the underlying periciliary fluid, minimizing thereby the drag on the mucus in the opposite direction. This asymmetry in the beat pattern is responsible for a net fluid flow in the direction of the effective stroke. In the airways, each mature ciliated cell may be covered with up to 200 cilia, with a surface density around 5–8 cilia/μm^2^ [6, 9]. Each cilium, approximately 6 μm long and of diameter around 0.2 μm, beats 12 to 15 times per second, resulting in a velocity of the mucus layer of approximately 1 mm per minute [10].

Defects in mucociliary clearance may result in chronic airway inflammation and infections causing injury and structural changes to the airway epithelium, leading to a variety of diseases like bronchiectasis and chronic sinusitis. Two main reasons may lead to impaired mucociliary clearance. The first one is related to alterations of the mucus properties, as in cystic fibrosis where the mucus become too thick and sticky to be moved properly by the cilia. The second one is linked to a dysfunction in ciliary motion or ciliary coordination. Such dysfunctions may be either inherited as in Primary Ciliary Dyskinesia (PCD) or acquired. Diagnosis of dyskinesia is difficult. For instance, in the diagnosis of PCD, a combination of transmission electronic microscopy, nasal nitric NO, genetic testing and use of high-speed video-microscopy is recommended [11].

*In situ* observation of ciliary beating and mucociliary clearance is almost impossible in patients at present stage, and one currently lacks a reliable and general method for evaluating mucociliary clearance in the clinical field. In the past, integrated assessment of mucociliary clearance was achieved through techniques using saccharine [12], a drop of blue marker [13], or clearance of radioactive tracers [14–17]. More recently, micro-optical coherence tomography was also proposed [18]. However, due to their various requirements (patient cooperation for the saccharine test, endoscopic examination, inhalation of radiopharmaceutical, …) these different methods are rarely used in the current clinical practice. Light microscopy observation of ciliated edge obtained by nasal or bronchial brushing is the most common method used to evaluate ciliary beating. This observation is often associated with High-Speed Video-Microscopy (HSVM) analysis [19–23] that quantifies only cilia motion and ciliary beat pattern. However, none of these tests provides information about the global efficiency of ciliary beating regarding mucociliary clearance.

This present study aims at a deeper understanding of the relationship between ciliary beat pattern, measured by ciliary beating analysis, and the induced motion of the surrounding fluid in order to assess the global efficiency of ciliary beating. An original method of micro-bead tracking (MBT) is presented where micro-beads act as markers of the flow generated by the cilia [24]. A numerical model is then developed (presented in detail in a companion paper [25]) based on envelope modeling approach, allowing us to simulate the flow generated by the cilia as in the MBT experiment. We finally propose a new and global index for characterizing the efficiency of the ciliary beating. One very appealing aspect of this index is that it does not require any modification of the present clinical practice of data collection (nasal or bronchial brushing).

## Materials and methods

### Experimental analysis

#### Patients

Studies of ciliary beating and MBT were performed in 11 consecutive patients referred to our diagnostic center. All patients were investigated because of chronic upper and/or lower respiratory tract infections, i.e., bronchitis and/or bronchiectasis and sinusitis. Patients were selected only after ruling out genetic disorder as cystic fibrosis or PCD. Informed consent was obtained from all patients, and this study was approved by the local Ethics Committee (Comité de Protection des Personnes Ile-de-France XI).

#### Digital high-speed video-microscopy

**Fig 1:**
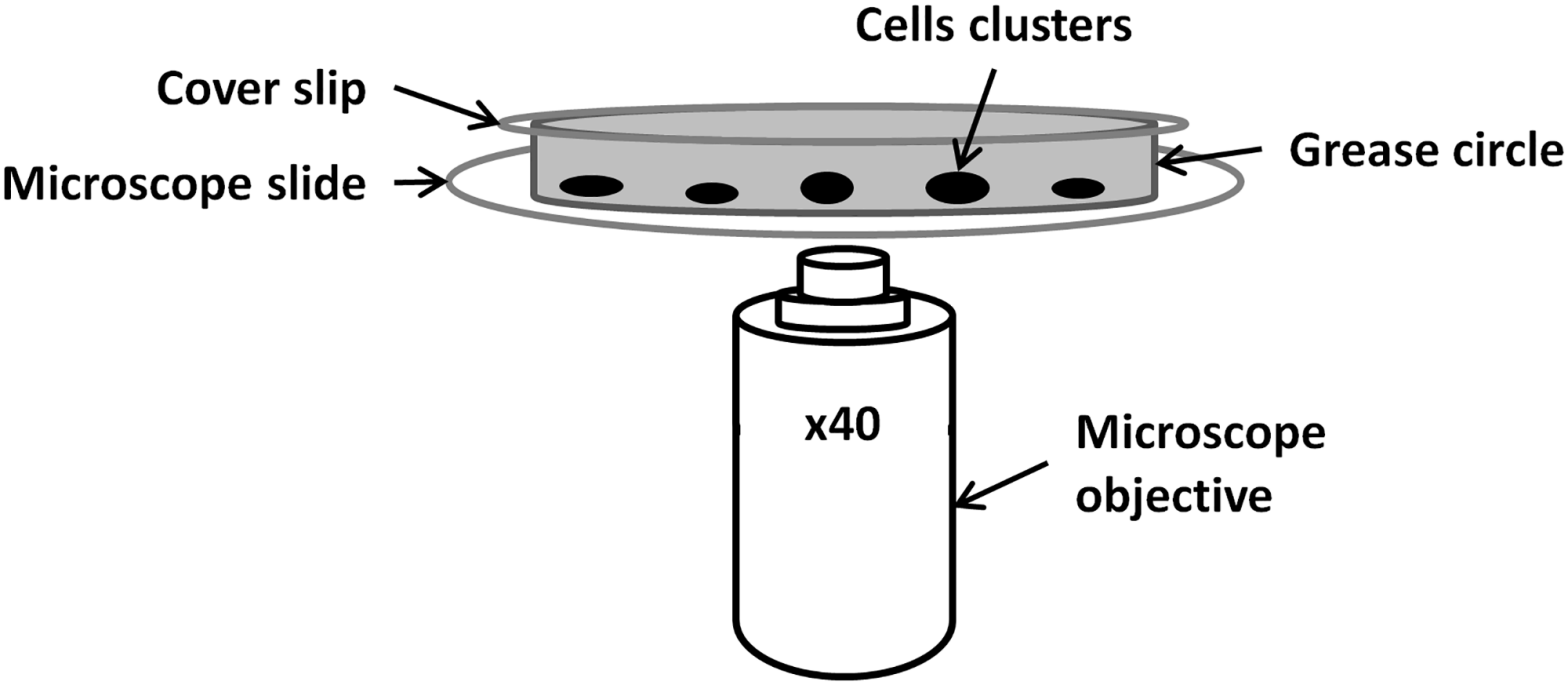
Scheme of experimental setup.

Ciliated samples were obtained by brushing the middle part of the inferior turbinate with a 2 mm cytology brush. Cells were suspended in a survival medium and examined within three hours. 20 μL of 4.5 μm polystyrene micro-beads at the concentration of 0.125%*w*/*v* were added to 80 μL of the medium. All observations were performed within 20 min, at controlled room temperature (20–25°C). We used an inverted microscope (with a LD condenser 0.35 in H position, i.e., without any phase contrast or differential interference contrast) in brightfield conditions associated with a ×40 objective. 100 μL of medium containing beating ciliated edges and microbeads in suspension are comprised between a microscope slide and a cover slide. The medium is delimited in the horizontal plane by a circle of grease of diameter approximately equal to 1 cm (Fig 1). Recorded ciliated edges are located in the horizontal focal plane of the microscope (so that observed cilia are horizontal). The “local plane” of the cell cluster, which corresponds to the cell-medium interface, is therefore essentially perpendicular to the substrate in the focal plane. The observed region lies at the intersection of the horizontal focal plane and the locally vertical cluster plane.

Cilia were recorded with a digital camera at a rate of 358 frames per second (brightness 0%, gamma 220, shutter 720 s, gain 1.160 dB). Each movie was composed of 1,800 frames with a definition of 256 × 192 pixels, each individual pixel being (0.32 × 0.32) μm^2^. All areas containing intact undisrupted ciliated epithelial edges larger than 50 μm, devoid of mucus, beating in the plane of the camera and close to micro-beads were recorded. As recommended in [26], isolated ciliated cells were excluded. Basically and except for the presence of microbeads, the high-speed video-microscopy procedure used here is equivalent (setup requirement and protocol) to the ones used by different groups for PCD diagnosis [19–21, 23].

#### Micro-bead tracking method

**Fig 2:**
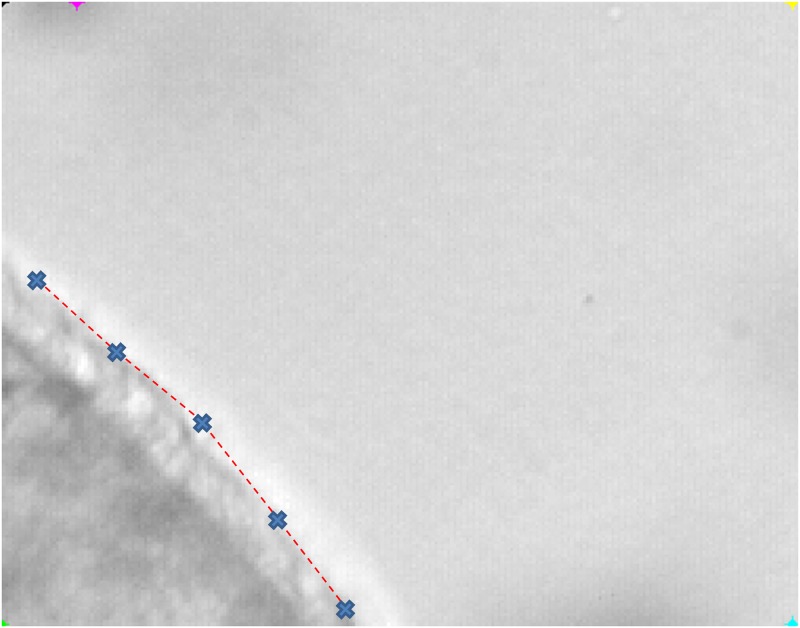
Determining the location of the ciliated edges. The operator places 5 points (blue crosses) which are the ends of 4 line segments (red dashed lines) defining the location of the cilia wall.

Locations of ciliated edges were determined using in-house software which reports positions of computer mouse clicks on paused video frames. Practically, the operator chooses 5 points delimiting 4 line segments. These segments define the location of the cilia wall (Fig 2). Micro-bead motions are tracked using an automatic image processing method programmed in Python language. First, the PINK Image Processing Library is used to threshold the frames, thus separating the micro-beads from the background. Secondly, micro-beads are labeled and their trajectories are plotted. Instantaneous micro-bead velocities, as well as their distance to the ciliated edge, are measured and saved in a database.

#### Analysis of ciliary beating

Ciliary beat frequency (CBF) is determined from movies in two different ways. In both methods, the operator defines a line by placing five points on the ciliated edge. In the first method, the variation of the mean grey level of this line during the movie is calculated. A Fast Fourier Transform (FFT) is performed on this signal, and the frequency of highest amplitude in the FFT power spectrum is taken as CBF. In the second method, video-kymography is used by stacking all lines obtained from each frame of a movie on the same graph. The beating period is measured on this graph, from which the ciliary beat frequency is deduced.

To characterize the ciliary beating amplitude (CBA), we used a simplified quantitative analysis of ciliary beat pattern previously described by Papon et al. [20]. In short, one individual cilium that can be followed during a complete beating cycle (excluding cilia whose tip ran out of the focal plane) is selected. Video sequences are played back frame by frame in order to determine three points characterizing the complete cycle of the cilium. These three points correspond to the position of the base of the cilium (P0) and the positions of the tip before the active and recovery strokes (P1 and P2, respectively). These measurements are used to determine the cilium length and the distance traveled by the cilium tip (i.e., the ciliary beating amplitude).

**Fig 3:**
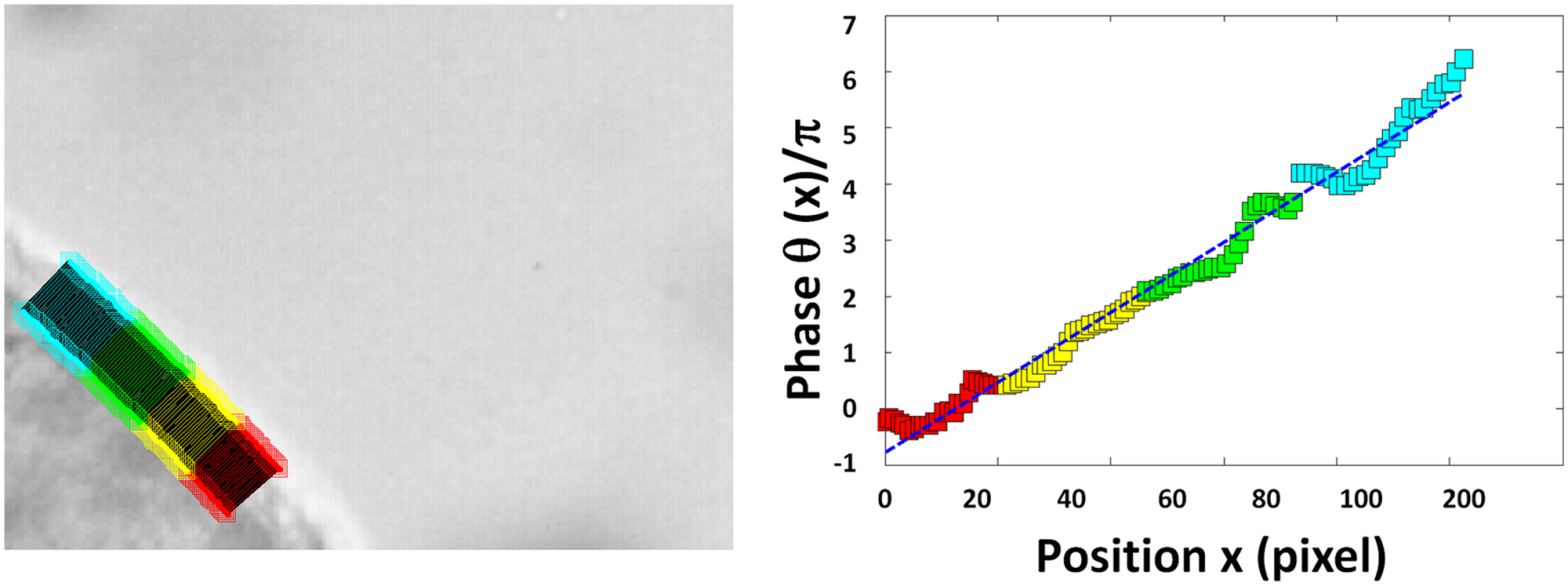
Example of wavelength determination. (Left) 104 line segments are placed along the cilia wall, perpendicular to it. (Right) The mean grey level of each segment oscillates in time. The corresponding phase each segment is plotted as a function of the curvilinear abscissa of the center of each line. A linear regression is performed on the phase-abscissa relationship, the slope of this regression directly giving the metachronal wavelength (blue dashed line).

The metachronal wavelength *λ* is obtained by inferring the phase variation of the main beat frequency along the ciliated edge. To this end, 104 line segments perpendicular to the cilia wall are defined (see Fig 3, left), each segment being made of 24 pixels. In each segment, the measured mean grey level oscillates cyclically in time. The phase of this variation, *θ*, is computed by a Fast Fourier Transform. In the case where this phase *θ*(*x*) depends linearly on the position *x* along the ciliated edge, a linear regression is used to determine the average slope *p*, hence the wavelength of the metachronal wave given by *λ* = 2*π*/*p* (Fig 3, right).

**Fig 4:**
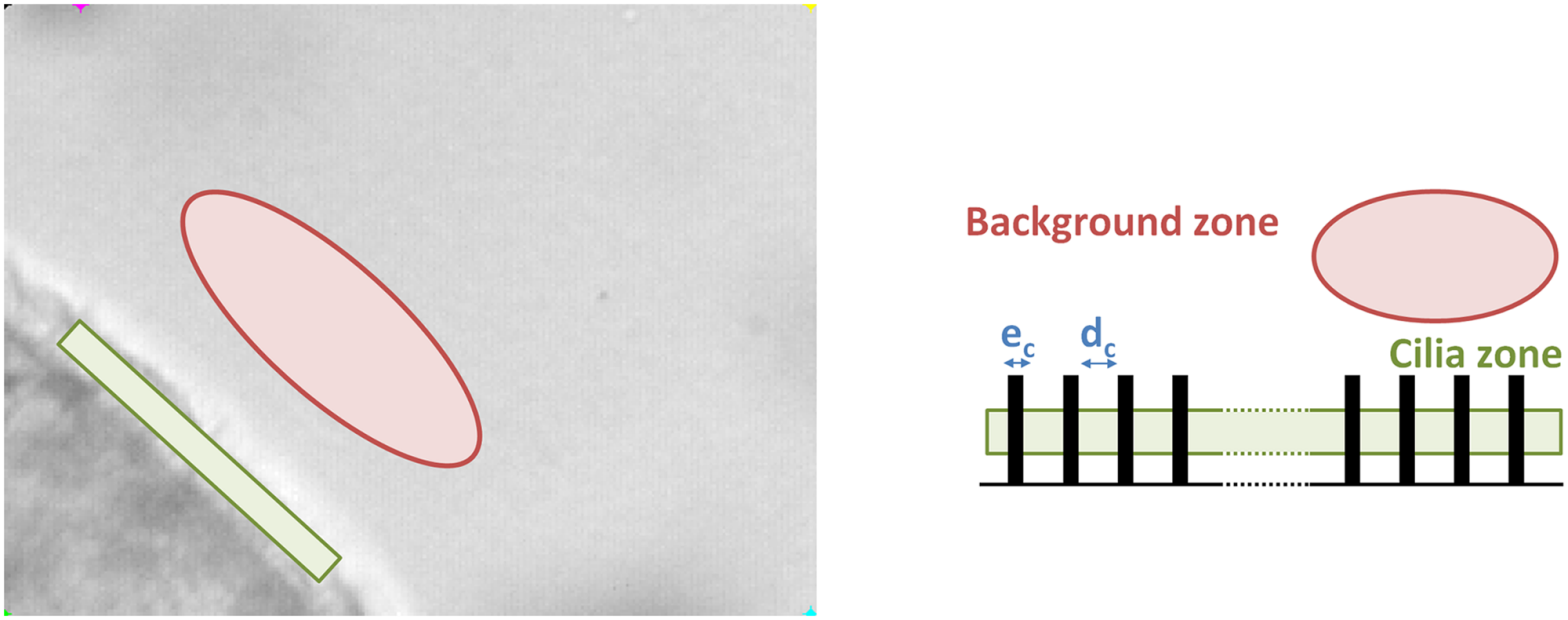
Measuring the cilia density. (Left) Two different regions are delimited on the real system, the green rectangle corresponds to the cilia region while the red ellipse corresponds to the background. (Right) Schematic representation of the same system: black rectangles represent the cilia, *e*_*c*_ is the cilium diameter and *d*_*c*_ is the typical distance between two cilia.

In addition, cilia density is estimated for each ciliated edge by comparing the mean grey level of the movie background with the grey level of the cilia area. In the image, cilia appear darker than the background (Fig 4, left). Cilia density is therefore calculated by an in-house software as the percentage of pixels belonging to the ciliated edge darker than the mean grey level of the background. This procedure allows us to define a characteristic length of the distance between two cilia (*d*_*c*_) and the fraction of area covered by the space without cilia (*f*_*c*_).

Let us consider an homogeneous distribution of cilia. In this case, the cilia density is given by:

$$\begin{array}{cc} \rho_{c} & =(\frac{\;\text{number}\;\text{of}\;\text{pixel}\;\leq \;\text{Mean}\;\text{Background}}{\text{number}\;\text{of}\;\text{pixel}}){|}_{\text{cilia}\;\text{zone}}\\ & =\frac{(e_{c}/\text{pixel}\;\text{size})\times \text{number}\;\text{of}\;\text{pixel}}{[(e_{c}+d_{c})/\text{pixel}\;\text{size}]\times \text{number}\;\text{of}\;\text{pixel}}=\frac{e_{c}}{e_{c}+d_{c}}, \end{array}$$ (1)

where *e*_*c*_ is the cilium diameter (0.2 μm). As such, what is called “cilia density” here is a relative measure of the cilia density obtained by averaging over a sufficient large number of individual pixels in the region of the ciliated edge. Let us note that (1 − *ρ*_*c*_) is in this case the fraction of area without cilia.

#### Data selection

Only the movies where the inputs required for ciliary beating simulation could be measured were retained (ciliary beat frequency, ciliary beating amplitude, metachronal wavelength and cilia density). These movies are the ones in which the cluster surface is essentially vertical so that the cilia edge located at the intersection of the cluster plane and the focal plane can clearly be observed. To remain within the assumptions of the model, we only selected micro-bead following straight trajectories close to a ciliated edge (i.e., not influenced by the external environment or other distant clusters). Finally, all analyses were performed by one single operator.

### Numerical model

**Fig 5:**
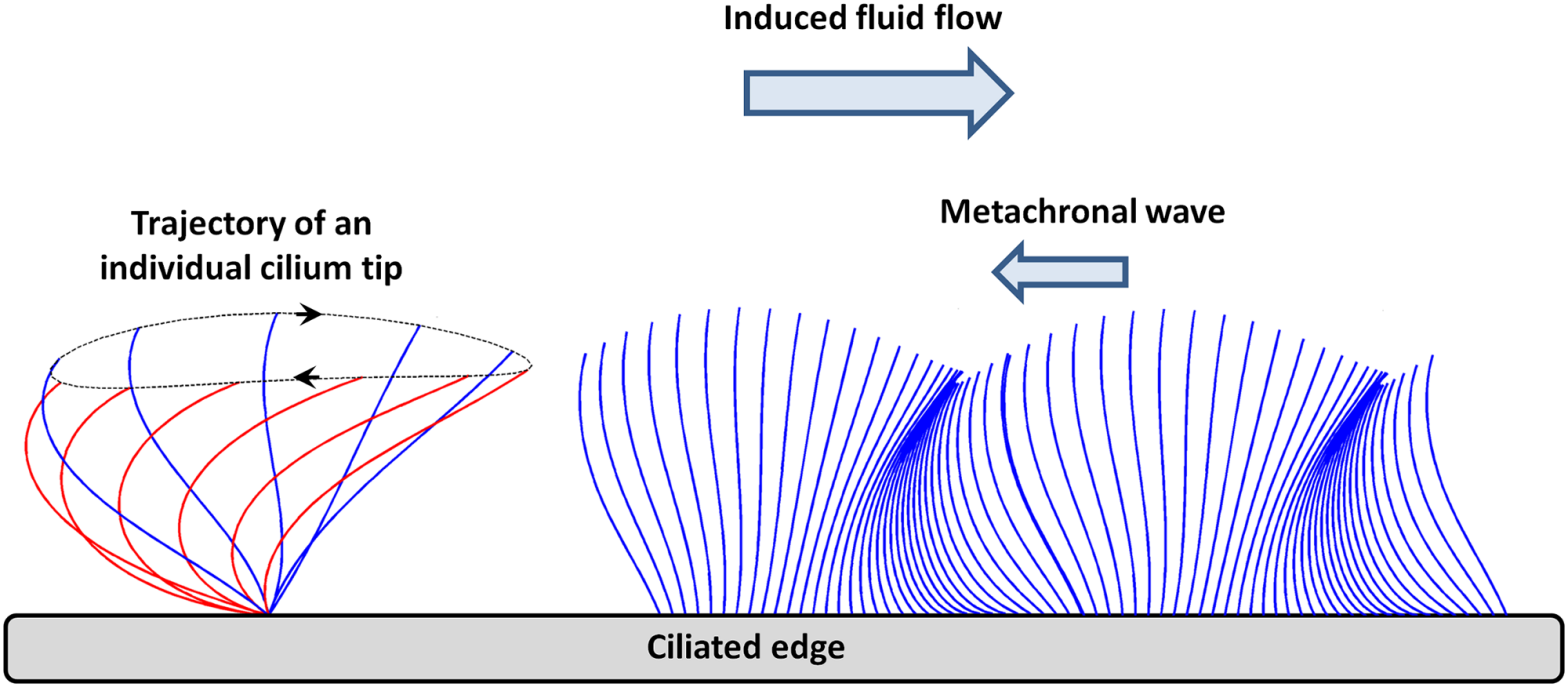
Schematic representation of the stroke of an individual cilium and the envelope model. (Left) The trajectory of the stroke cycle is assumed to follow an elliptic motion. (Right) Representation of the envelope model covering the cilia layer and the propagation of the metachronal wave. (Inspired by Velez-Cordero et al. [28]).

We present here a brief overview of the two-dimensional model describing the system composed of the cilia, the surrounding fluid, and the micro-beads. The entire model is extensively detailed in [25]. Inspired by [27], we propose an approach in which the momentum transfer from *discrete cilia* to the fluid (induced by the ciliary beating) is modeled through an appropriate continuous boundary condition. The ciliated edge is chosen parallel to the *x* axis, the ciliary tip being located around *y* = 0 on average. Each cilium is assumed to undergo a periodic elliptic motion (see Fig 5, left). Taking the limit of a continuous cilia distribution, the cilia array is simplified as an undulating surface that covers the cilia layer, ignoring the details of the sub-layer dynamics, see Fig 5, right (inspired by Velez-Cordero et al. [28]).

**Fig 6:**
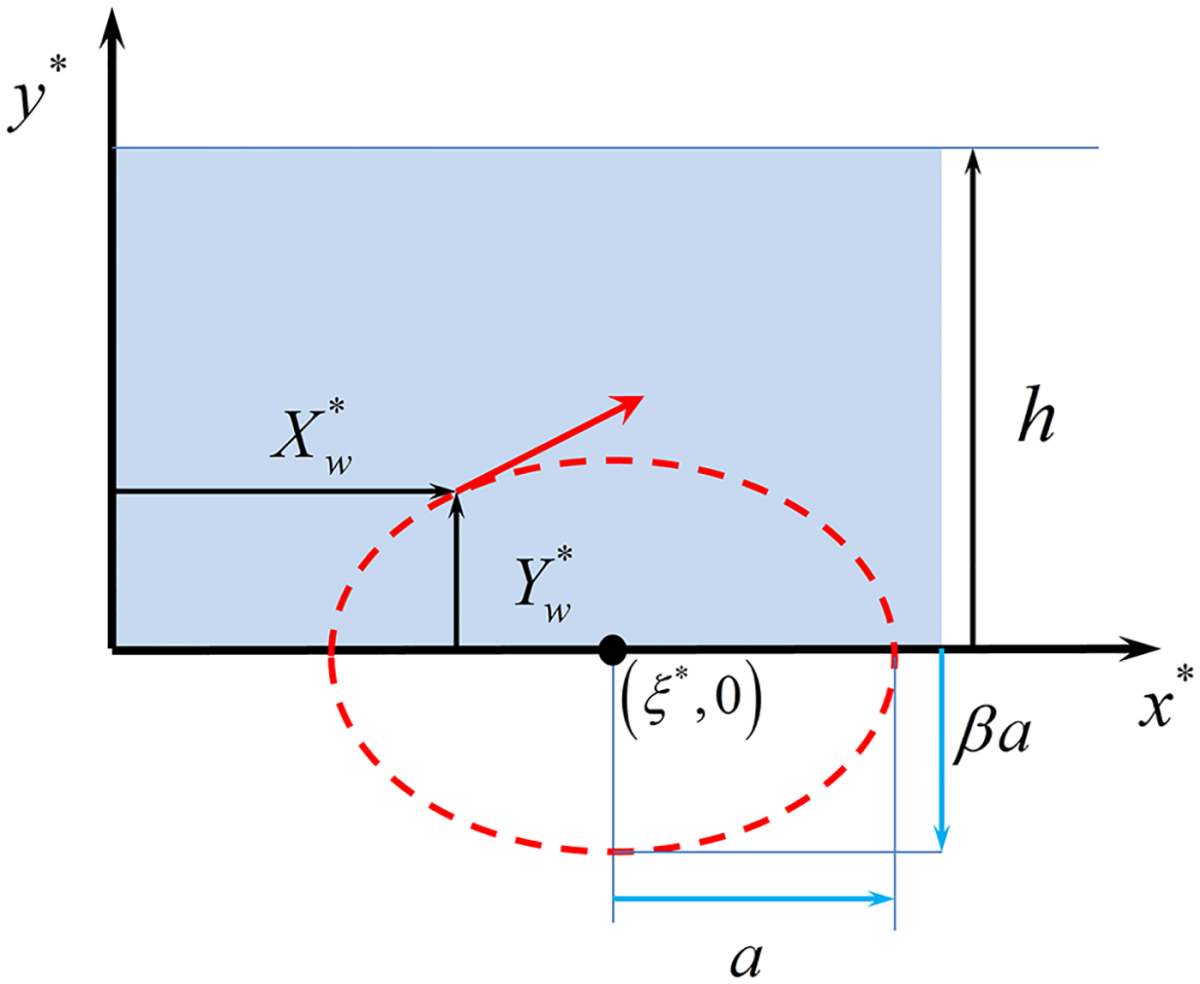
Schematic elliptic motion of an individual ciliary tip.

The tip of a cilium located at the horizontal coordinate ξ* is assumed to follow a periodic elliptic trajectory centered in (ξ*, 0) during each elementary beat (Fig 6). The ‘*’ notation is used here to represent dimensional quantities, for consistency reason with [25]. At time t*, the tip coordinates $\left(X_{w}^{*},Y_{w}^{*}\right)$ satisfy

$$\left\{ \begin{array}{l} X_{w}^{*}=\xi^{*}-a\text{ cos}\left(\omega t^{*}\right)\\ Y_{w}^{*}=\beta a\text{ sin}\left(\omega t^{*}\right) \end{array}\right.$$ (2)

where *β* is a function of the ellipse eccentricity, 2*βa* is its minor axis in the y* direction, and 2*a* its major axis in the x* direction. For *β* > 0, the tip orbits clockwise, while for *β* < 0, the tip orbits counterclockwise.

In this picture, the metachronal wave is materialized by prescribing the motion of the envelope. This envelope is then used as a boundary condition to compute the fluid velocity field dominated by viscous forces, inside a channel of height *h* (see Fig 6). The fluid is assumed to be stagnant above this height. Indeed, the parameter *h* essentially summarizes in our 2D model (see companion paper) the effect of the external environment of the clump, this environment imposing a limit to the spatial extension of the cilia-induced flow field. This fact explains why this parameter *h* cannot be estimated a priori and has to be measured as an external free parameter.

The trajectories of micro-beads in this velocity field are calculated by solving the equation of motion with Stokes drag. This theoretical model provides us with a prediction of the effective speed of micro-beads as a function of their average altitude above the ciliated edge. This velocity profile is found to follow essentially a parabolic profile as a function of the height above the cilia wall. Finally, the velocity of the micro-beads extrapolated at the cilia wall gives a direct estimate of the shear stress exerted on the fluid, this index being proposed as an index of the ciliary beating efficiency [25].

#### Simulation of micro-beads effective velocity

The bead inertia in the fluid flow is characterized by their Stokes number, *St*_*k*_. For particles of mass *m* and radius *R* traveling through a fluid of viscosity *μ*, of typical velocity *U* and of typical length *L*, this number is defined as:

$$\begin{array}{c} St_{k}=\frac{mU}{6\pi R\mu L} \end{array}$$ (3)

For spherical particles of radius *R* and density *ρ*_*b*_ in an oscillatory flow of pulsation *ω*, this number also reads:

$$\begin{array}{c} St_{k}=\frac{\frac{4}{3}\pi R^{3}\rho_{b}\;\omega }{6\pi R\mu }=\frac{2}{9}\frac{R^{2}\rho_{b}\omega }{\mu } \end{array}$$ (4)

The micro-beads are about 4.5 μm diameter, and made out of polystyrene of density *ρ*_*b*_ of order 1 g.cm^−3^. At 10 Hz in water, the corresponding Stokes number is therefore about 10^−4^, which means that the beads can be considered as massless tracers. Their velocity can be assumed to be permanently equal to the fluid velocity at the same location.

For each micro-bead entering the simulation window at *x* = 0 and a given altitude *y*_0_, the effective speed is computed as:

$$\begin{array}{c} V_{\text{eff}}\left(y_{0}\right)=\frac{L_{w}}{\tau (y_{0})}=\frac{L_{w}}{\oint_{T_{y_{0}}}dt}=\frac{L_{w}}{\oint_{T_{y_{0}}}\frac{ds}{∥\vec{u}\left(s\right)∥}}\;, \end{array}$$ (5)

where *L*_*w*_ is the length of the observation window, *τ*(*y*_0_) is the crossing time of the micro-bead entering at (0, *y*_0_), and $T_{y_{0}}$ is the trajectory followed by this micro-bead. During each elementary step of this trajectory, the infinitesimal duration is $dt=ds/∥\vec{u}\left(s\right)∥$, $\vec{u}$ being the fluid velocity at curvilinear abscissa *s* of the trajectory. The effective speed *V*_*eff*_ corresponds to the quantity measured in our experiments.

## Results

### Micro-bead tracking method

78 movies were recorded, corresponding to a total of 24 ciliated edges. From these movies, the trajectories of 195 micro-beads were retained. Three examples of MBT movie are displayed online (see supporting information).

**Fig 7:**
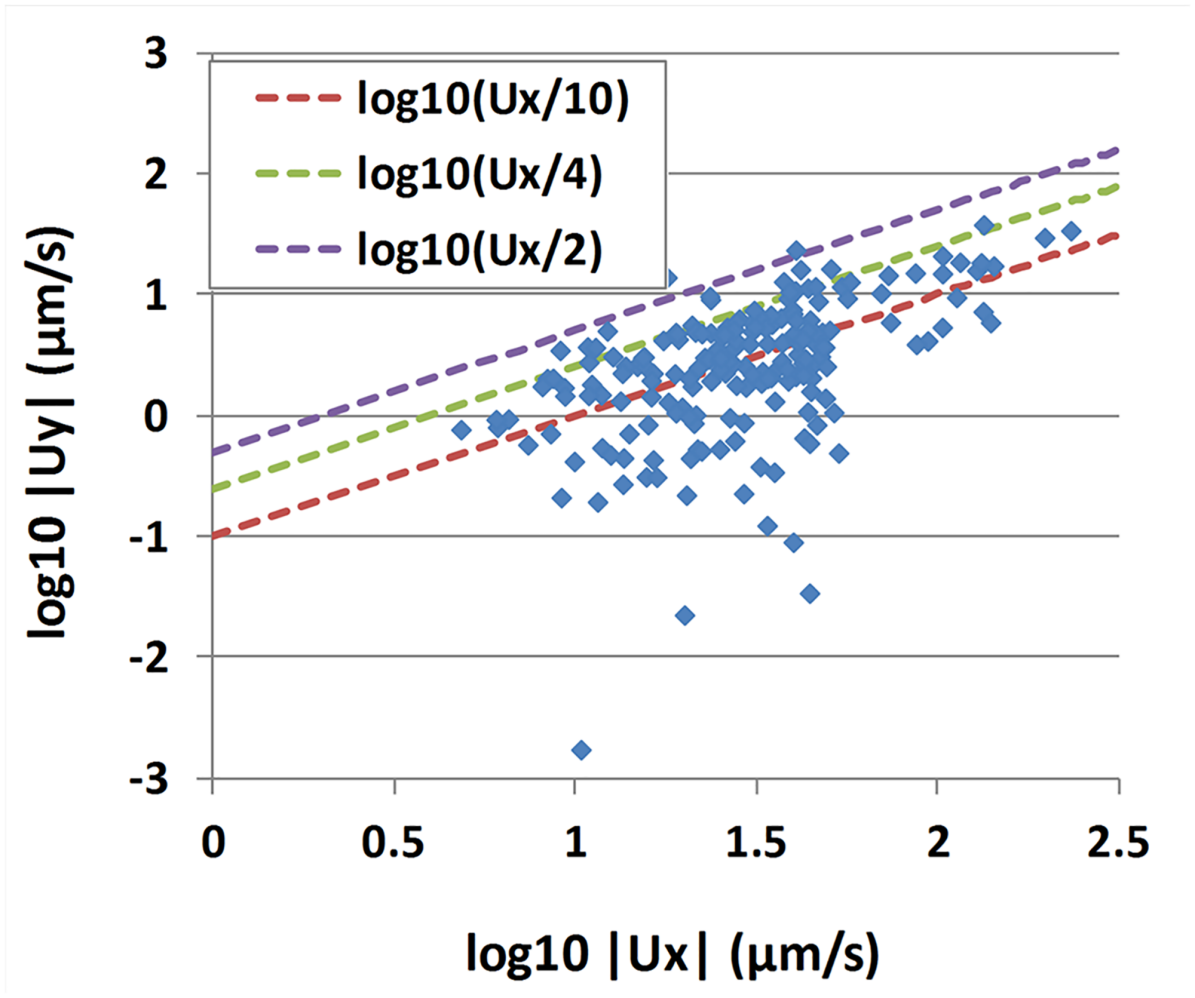
Vertical velocity component versus horizontal velocity component. Each square corresponds to one of the 195 micro-beads measured. Red, green and purple dashed lines correspond respectively to |*u*_*y*_| = |*u*_*x*_|/10, |*u*_*y*_| = |*u*_*x*_|/4 and |*u*_*y*_| = |*u*_*x*_|/2.

Micro-beads velocities are essentially oriented along the x* direction (parallel to the ciliated edge), as can be seen in Fig 7. Only 4% of the micro-beads exhibit a vertical velocity component larger than 25% of the horizontal component. Moreover, this vertical component is about equally distributed among positive and negative values (98 vs 97). This result suggests that the bead velocities can be modeled in a good approximation as parallel to the ciliated edge.

**Fig 8:**
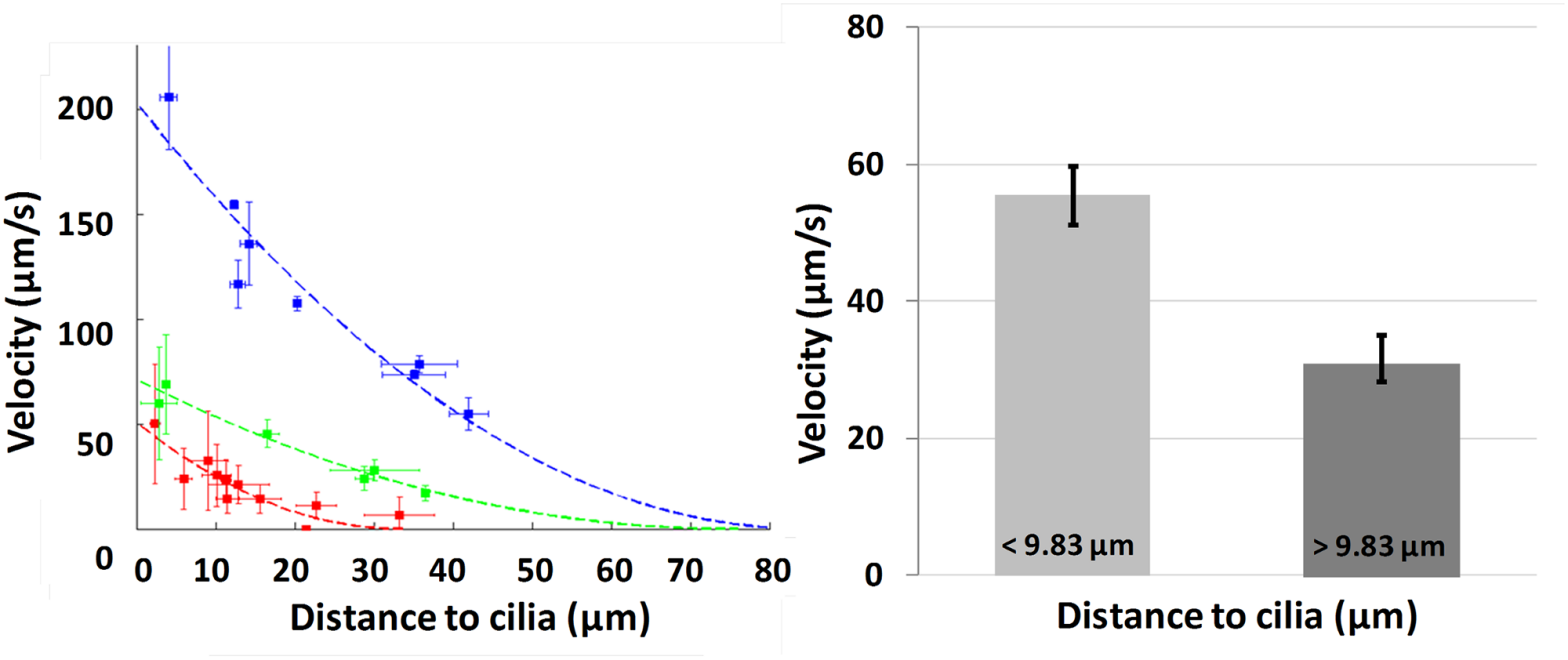
Micro-bead velocities versus distance to the ciliated edge. (Left) Examples of mean velocity obtained in 25 micro-beads in 3 ciliated edges (blue, green and red). Each square corresponds to one micro-bead. Horizontal and vertical error bars display for each bead the standard deviation of the distance to cilia and velocity, respectively. Dashed lines are the parabolic regression on each ciliated edges. (Right) The 195 measured micro-beads are presented into 2 groups of equal sizes, according to their distance to the ciliated edge. Bars exhibit a significant velocity difference (*p* < 0.05). Error bars represented the standard error of the mean (SEM).

Micro-bead velocities range from 0.0 to 253.8 μm.s^-1^ (mean = 42.2 μm.s^-1^, std = 35.0 μm.s^-1^). This wide variation is explained by the spread of the distances of the micro-beads to the ciliated edge which range from 0.3 to 70.9 μm (mean = 12.7 μm; std = 11.1 μm). Indeed, as observed in Fig 8, left which presents micro-bead velocity measurements on three different ciliated edges, velocities appear to be strongly correlated to these distances, the fastest micro-beads being the ones closest to the ciliated edge. To confirm this observation, velocities of all micro-beads were separated into two groups, respectively above and below to the median value of the distance (Fig 8, right). The comparison between the two groups was performed with a statistical software package using non-parametric tests (Mann-Whitney U-test). A *p* value <0.05 was considered significant. Here again, one can distinctly observe a clear link between the value of the micro-bead velocity and its distance to the ciliated edge.

### Correlation between micro-bead velocity and other ciliary beating measurements

In order to assess the parameters that influence significantly the micro-bead velocity, a multiple linear regression analysis was performed between the values of the micro-bead velocities (the *dependent* variable in the regression) and five *exploratory* variables, 4 of which are determined from measurements of ciliary beating (CBF, CBA, *λ*, *ρ*_*c*_), and the fifth being the distance *y*_0_ to the cilia. The resulting multiple regression equation of micro-bead velocities is given by:

$$\begin{array}{cc} V_{\text{eff}}\left(y_{0}\right)=\; & \left(6.96\times \text{CBF}\right)+\left(230.6\times \rho_{c}\right)+\left(11.95\times \text{CBA}\right)-\left(1.22\times y_{0}\right)\\ & -(0.83\times \lambda )-243.35\;, \end{array}$$ (6)

with a coefficient of determination equal to 0.68 and a probability smaller than 10^−6^ (F-test). Moreover, all probabilities associated to regression coefficients (t-test) are smaller than 10^−6^, except for the wavelength (*p* = 0.048). One can remark that this regression implicitly assumes a linear dependency on the exploratory variables, while a further and finer analysis using our mathematical model reveals a more complex behavior, as for instance the micro-bead velocity profile above the edge which is shown in fact to be essentially parabolic.

### Comparison between experimental measurement and numerical model

#### Input of the numerical model

To run simulations and compare their results with our MBT experiments, the numerical model presented in [25] requires 5 inputs: CBF, CBA, metachronal wavelength *λ*, distance *h*, and the slip length *ϕ*. CBF, CBA, and *λ* are directly measured by a microscopic analysis of the ciliary beating (see above). The distance *h* has to be determined by fitting the parabolic velocity profile. Finally, the slip length *ϕ* is shown to be directly correlated to the cilia density.

*Distance h*. The mathematical model predicts that the micro-bead velocity exhibits a parabolic profile along *y* [25]. The distance *h* between the cilia wall and the region of stagnant fluid is read as the *x*-intercept of the parabolic fit of the measured velocity profile.

**Fig 9:**
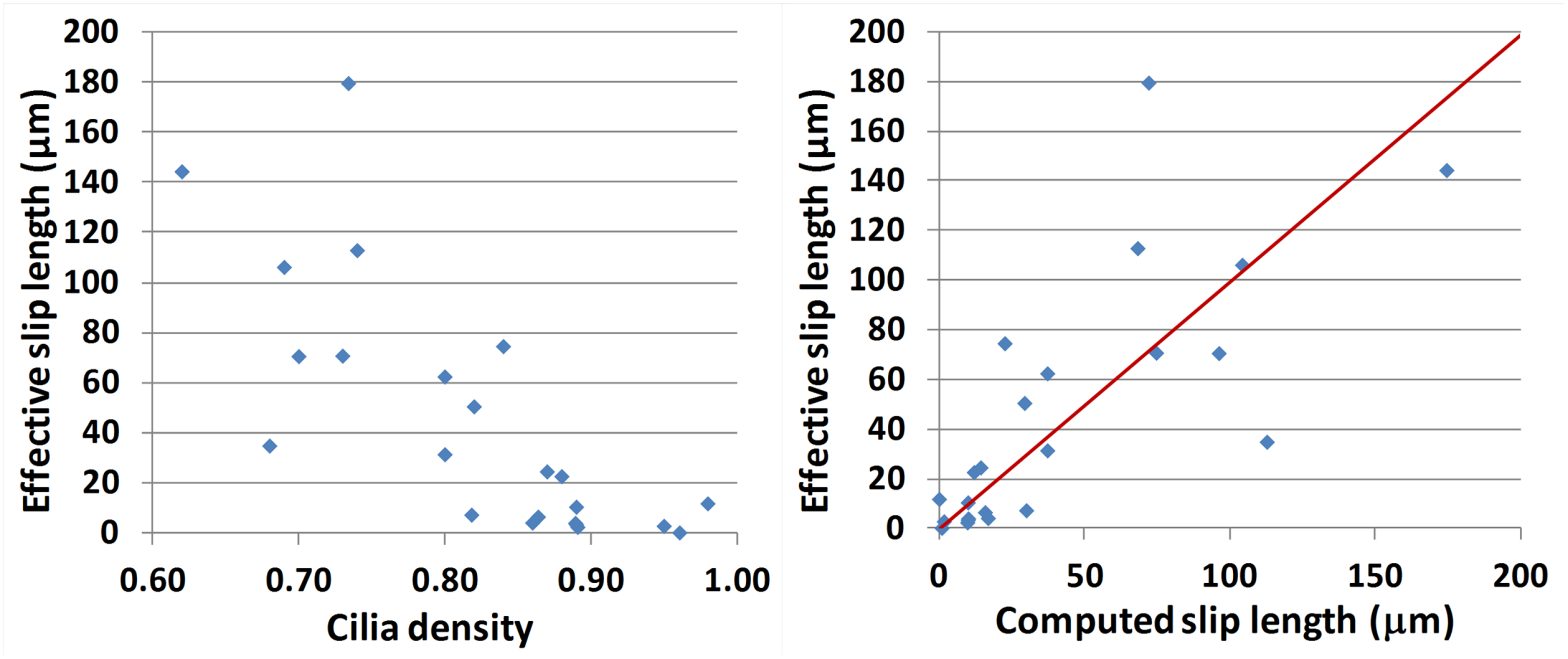
Effective porosity parameter *ϕ*. (Left) The effective slip length *ϕ*, obtained in each one of the 24 ciliated edges by fitting the measured micro-bead velocity with a parabola, plotted against cilia density. (Right) Effective slip length *ϕ* fitted from micro-bead measurements plotted against the effective slip length computed from the cilia density using Eq 8. The red line corresponds to the linear regression.

*Slip length ϕ*. The fifth and last parameter required as input to the numerical model is the slip length *ϕ* which cannot be directly retrieved from a microscopic measurement at the cilia wall. This parameter accounts for the partial momentum transfer between the wall and the fluid, due to the non continuous coverage of the cilia, through the following boundary condition:

$$\begin{array}{c} \vec{u}-\phi \frac{\partial \vec{u}}{\partial y}{|}_{\left(x,y_{w},t\right)}={\vec{u}}_{w}\left(x,t\right)\;, \end{array}$$ (7)

where $\vec{u}$ is the velocity and ${\vec{u}}_{w}\left(x,t\right)$ is the wall velocity. This condition is analogous to that of a fluid flow next to a porous wall, where the presence of pores reduces the transfer of momentum between the wall and the fluid [29, 30]. In this situation, *ϕ* can be understood an *effective slip length* [31]. The parameter *ϕ* can be retrieved from MBT experiments by fitting the measured micro-bead velocity profile above the cilia wall with a parabola. If *ϕ* = 0, one recovers a non slip boundary condition, while *ϕ* → +∞ corresponds to a perfect sliding condition. Sbragalia, *et al*. [32] showed that the effective slip length produced by a solid no-slip boundary with a distribution of free slip area was proportional to the product of the length scale of free slip by the fraction of surface covered by them. In our problem, if we assume that the tips of cilia are “no-slip” while the spaces between cilia are “free slip”, we expect the value of *ϕ* (extracted from the aforementioned fitting procedure) to be directly proportional to (1 − *ρ*_*c*_)^2^/*ρ*_*c*_ where *ρ*_*c*_ is the cilia density directly estimated from a grey level measurement. As shown in Fig 9, left, *ϕ* and *ρ*_*c*_ appear closely related. This correlation shows that a sparse density of cilia is a main determinant for a poor momentum transfer to the fluid. A simple regression method leads to the following analytic relationship between these two parameters *ϕ* and *ρ*_*c*_:

$$\begin{array}{c} \phi \approx \phi_{\text{calc}}\left(\rho_{c}\right)=\Lambda \frac{{\left(1-\rho_{c}\right)}^{2}}{\rho_{c}}\;\text{with}\;\Lambda =750\;\mu \text{m} \end{array}$$ (8)

Fig 9, right, displays a plot of the effective slip length *ϕ* extracted from the fitting procedure and the calculated slip length *ϕ*_*calc*_ obtained from the formula in Eq 8. Except for a couple outliers, most values appear to be very close.

#### Micro-bead velocities

**Fig 10:**
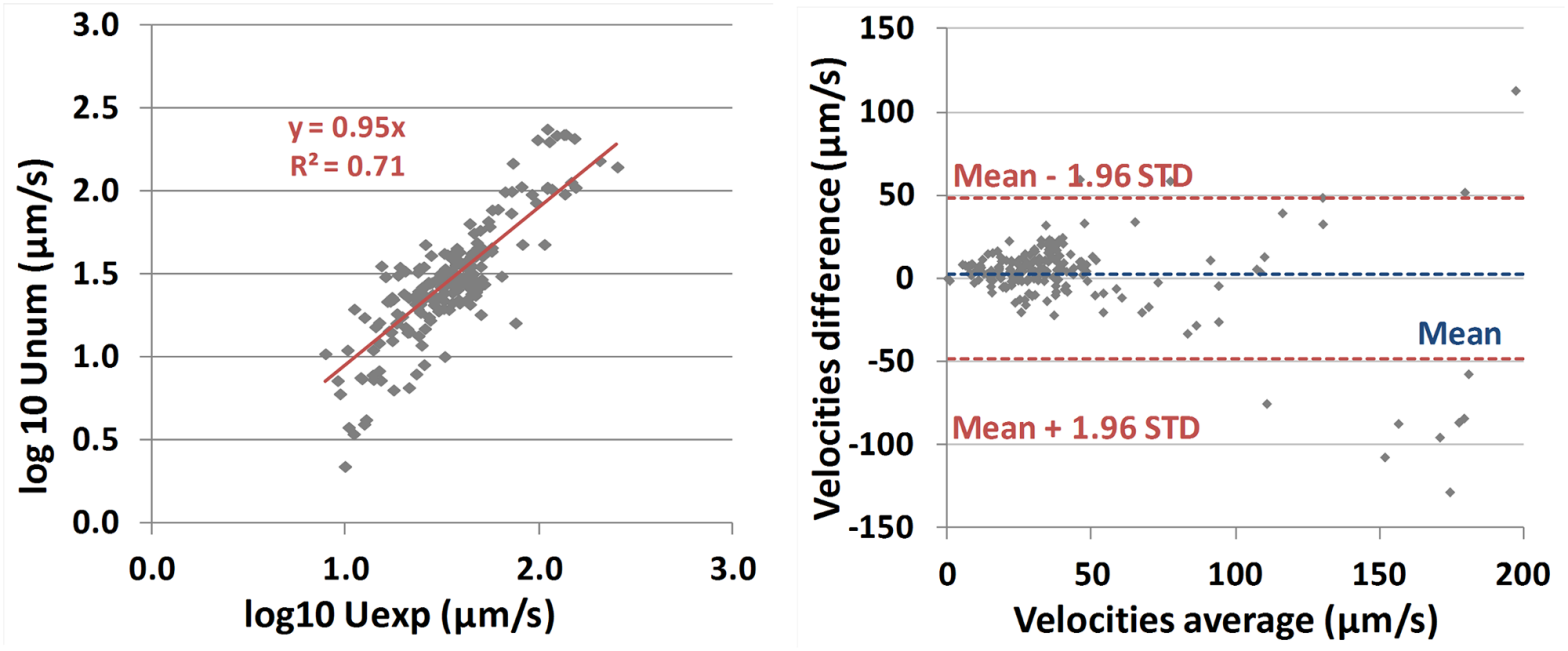
Comparison between measured and simulated micro-bead velocities. (Left) Experimental velocities against simulated velocities in the 195 measured microbeads. The red line corresponds to *y* = *x*. (Right) Bland-Altman plot. The blue dashed line corresponds to the mean value while the red dashed lines correspond to the mean value ± 1.96 standard deviation.

We now compare micro-bead velocities measured by MBT with numerical simulations using the 4 input parameters deduced from the analysis of ciliary beating: CBF, CBA, metachronal wavelength *λ*, and *ϕ*_*calc*_ (this last value being deduced from the measured cilia density). We add to these measured parameters the distance *h* fitted from MBT experiment. In Fig 10, left, the velocities *V*_*b*_ measured by MBT are plotted against the velocities *V*_*eff*_ predicted by the numerical model. Each point corresponds to one micro-bead. The correlation coefficient *R*^2^ between cilia density and slip length is found to be equal to 0.71. In Fig 10, right, the Bland-Altman plot displays the difference between the two velocities against their average, i.e., each point corresponds to $\left(\frac{V_{b}+V_{\text{eff}}}{2},V_{b}-V_{\text{eff}}\right)$. Again, experimental and numerical velocities are found to be in close agreement, proving that our model faithfully captures the relationship between fluid velocity and the microscopic measurements of cilia motion.

### Ciliary beating efficiency index

The shear stress *τ*_*w*_ exerted by the cilia wall on the fluid is given by (see [25]):

$$\begin{array}{c} \tau_{w}=\frac{2\mu }{h}U_{w}^{*} \end{array}$$ (9)

where $U_{w}^{*}$ is the extrapolated velocity at the ciliated wall, *h* is the horizontal intercept (vanishing velocity) of the fitted parabolic profile, and *μ* is the dynamic viscosity of the fluid. We propose to use this force *τ*_*w*_ as an index for assessing the efficiency of the ciliary beating. MBT measurements enable us to retrieve $U_{w}^{*}$ and *h*, and therefore to compute the shear stress.

In Table 1 we report the results of the analysis performed on 11 patients. For each patient, one or several ciliated edges have been analyzed. For each edge, the number of tracked micro-beads is reported, followed by measured values of the input parameters of the model. The last 3 columns show the values of *h* and $U_{w}^{*}$, retrieved from the parabolic profiles, and the value of the deduced shear stress.

**Table 1:** Value of efficiency index for all ciliated edges.

Patient №	*n*	CBF (Hz)	CBA (μm)	*λ* (μm)	*ρ*_*c*_ (%)	*h* (μm)	$U_{w}^{*}$ (μm.s^-1^)	*τ*_*w*_ (mPa)	mean ± std dev.
1	8	14.0	7.5	13.0	84	87.5	138.3	3.2	5.8±4.1
	4	15.0	6.8	14.6	86	52.6	240.9	9.2	
	5	13.5	7.5	19.8	89	47.4	224.8	9.5	
	5	13.6	4.0	28.0	82	70.9	49.4	1.4	
2	3	5.3	7.0	10.8	73	7.0	27.6	0.7	2.5±1.6
	3	5.6	6.6	11.3	80	32.9	46.9	2.9	
	9	6.3	7.3	11.0	68	28.9	55.2	3.8	
3	6	14.0	5.4	10.5	69	53.0	51.3	1.9	1.9
4	4	11.5	7.5	12.0	74	66.3	76.8	2.3	2.3
5	3	7.3	7.8	16.0	58	82.1	24.0	0.6	2.0±1.3
	3	9.4	7.0	15.5	80	91.5	80.1	1.8	
	3	4.9	8.4	17.3	88	53.5	64.7	2.4	
	4	5.6	6.9	13.3	95	52.8	106.5	4.0	
	3	8.1	6.7	15.4	82	139.7	85.4	1.2	
6	4	3.7	6.2	16.7	89	34.2	33.6	2.0	2.0
7	4	7.7	7.2	12.0	70	42.6	49.3	2.3	2.3
8	3	3.6	6.3	17.7	98	40.1	32.3	1.6	1.6
9	12	7.7	8.8	12.1	62	60.0	51.7	1.7	1.9±0.3
	5	6.6	7.0	10.3	73	52.3	54.4	2.1	
10	3	6.7	6.5	10.4	87	28.6	60.2	4.2	4.2
11	56	3.6	4.7	10.2	96	51.2	58.9	2.3	2.1±0.3
	11	3.5	5.3	12.6	89	48.6	45.6	1.9	
	6	4.9	4.8	18.5	89	50.5	44.3	1.8	
	28	6.0	5.2	16.7	86	45.4	56.4	2.5	

№ corresponds to the patient number; *n* is the number of micro-beads per ciliated edges; CBF is the ciliary beat frequency; CBA is the ciliary beat amplitude; *λ* is the metachronal wavelength; *h* is the distance fitted from the micro-bead velocity profile; *ρ*_*c*_ is the cilia density; $U_{w}^{*}$ is the extrapolated velocity at the ciliated wall through MBT; *τ*_*w*_ is the shear stress exerted by the cilia wall on the fluid (see Eq 9). When several ciliated edges are measured for the same patient, the standard deviation of *τ*_*w*_ is computed.

## Discussion

The experimental MBT results (see Fig 10, left and right), allowed us to validate the numerical model. The model predicts that the micro-bead velocity is an increasing function of CBA and CBF while it decreases with *λ* and *ϕ* (the last dependency means that micro-bead velocity increases with *ρ*_*c*_). These behaviors are consistent with the various signs of the regression coefficients of Eq 6 deduced from MBT experiments.

The good agreement observed between the numerical model and experimental MBT results led us to propose the steady component of the shear stress exerted by the cilia wall on the fluid as a new index of the ciliary beating efficiency. To our knowledge, there exist very few evaluations of the shear stress exerted by the respiratory cilia in the literature. If we assume a cilia density per unit area of 5 cilia/μm^2^ [9], we find a force per cilium equal to 0.013±0.009 pN. Such a value is much lower (at least 3 orders of magnitude) than the ones reported in a study on human bronchial epithelial cell culture [9], or in a study on culture grown from frog esophagus [33]. However, in these two last cases, the measured quantity was an oscillating force and not the steady component resulting from the entire beating as in our study. This suggests that these two types of measurement should not be directly compared. Moreover, the shear stress we find is of the same order of magnitude than the shear stress induced by the so-called Couette flow between two parallel flat plates:

$$\begin{array}{c} \tau_{\text{Couette}}=\frac{\mu }{e}V\;, \end{array}$$ (10)

where *e* and *V* are the distance and the relative velocity between the two plates, respectively. If we put on a par *e* with *h*, and *V* with $U_{w}^{*}$, respectively, one finds the formula for the two stresses to be very similar. The factor 2 observed in our mode originates from the difference of velocity profiles (linear for a Couette flow, parabolic in our case).

The parameter *β*, i.e., the ratio between minor and major axis is very difficult to measure on a large part of our movies. We decided to set *β* at 0.14 for all simulations as it was the mean value observed on our movies. The measured values of all other parameters obtained by ciliary analysis in this study were consistent with the literature. As an example, our values of metachronal wavelength fall in the same range than the few numbers that can be found in the literature for other cellular models (*paramecium* [34, 35], frog oesophagius [36] or rabbit trachea [5]).

In contrast with other analyses of the ciliary beating [20], or evaluations of the beat pattern [19], *τ*_*w*_ provides a direct estimation of the force that is potentially applied by the ciliated epithelium on the surrounding fluid, hence on the mucus. As such, it appears as a very good candidate for a global index of the potential ciliary beat efficiency. It encompasses the beating of the ciliated edge as a whole rather than focusing on an individual cilium. In comparable experimental conditions (here the cell survival medium at room temperature), *τ*_*w*_ allows us to compare the ciliary beat efficiency of different patients with each other. Moreover, this index that can be easily obtained from a MBT experiment via a simple parabolic fitting requires neither a subjective interpretation by an operator, nor a specific procedure in terms of human data sampling, but only a classical nasal (or bronchial) brushing.

Low values of *τ*_*w*_, corresponding to a low ciliary beat efficiency can be explained by several distinct causes: ciliary beating parameters alterations (CBF, CBA, metachronal wavelength), reduced cilia density, or a loss of coordination between cilia. Indeed our model assumes, via the metachronal wave, a perfect cilia coordination as well as a pure rectilinear geometry of the edge. As a consequence, high values of *τ*_*w*_ are expected to correspond to well coordinated cilia without degradation of ciliary beating parameters (CBF, CBA, metachronal wavelength). However, for low values of *τ*_*w*_, a ciliary beat pattern analysis may be required to find the reason of a degraded efficiency (ciliary beating parameters, loss of coordination, …).

Table 1 displays values of *τ*_*w*_ measured in several patients. These values exhibit a relatively high intra-patient heterogeneity which is not surprising as it was already observed in other characteristics of the ciliary beating, such as the “the distance traveled by the cilium tip in one second weighted by the percentage of beating ciliated edges” [20]. Despite its intra-patient variability, this distance is able to discriminate non-PCD from PCD patients with a specificity and sensitivity above 0.95 [20]. Similarly, using *τ*_*w*_ as a screening index in clinical studies would probably require analyzing several edges per patient.

Interestingly, the patient exhibiting the highest values of *τ*_*w*_ (patient №1, see Table 1) seems to be also the patient with the most normal clinical state. To this date, it is difficult to pinpoint a precise threshold of *τ*_*w*_ that would allow one to discriminate between clinically healthy and pathological edges. Determining this threshold would require a clinical study including a larger cohort of control patients compared to patients with well defined pathological ciliary beating phenotype, a study far beyond the scope of the present study.

Ciliary beating parameters (CBF, CBA, pattern, …) depend on the characteristics of the fluid at least in part. In our MBT experiments, cilia are beating in a fluid whose physical properties are very similar to water. Such conditions are far from the real conditions of airways coated with mucus. One may wonder how the modification of the surrounding fluid influences cilia beating. Several numerical studies have investigated the way viscosity influences ciliary beating and the difference between a Newtonian vs. non Newtonian surrounding fluid. Jayathilake *et al*. have explored numerically the effect of an increased PCL viscosity on the motion of cilia embedded, and have found that, for a given beating frequency (set around 10 Hz, consistent with our measurements), the velocities of the induced PCL flow were almost unaffected by a 5-fold increase of the viscosity [37]. Other models taking into account the thermodynamic characteristic of the mucus (generalized Newtonian fluid, …) can be found in the literature [28]. More recently, Sedaghat *et al*. have studied in detail the two layer structure (PCL and mucus), PCL being modeled as a Newtonian fluid and mucus as a viscoelastic fluid [38]. Their study showed firstly that mucus viscosity has a very limited effect on the mucus flow, due to the dominating part of the elastic part in the viscosity, and secondly that ciliary beating frequency plays a major role in the mucus velocity. These results were confirmed by very recent 3D numerical simulations [39]. In these works, the beating frequency was identified as a major determinant of the mucus flow, before the mucus viscosity itself. However, in all simulations, this frequency is always a prescribed parameter whereas in reality it results from a delicate force and momentum balance in the fluid-structure interaction. As a consequence, it is very complicated to really assess the net influence of mucus rheological properties on cilia beating, considering the extreme difficulty to reproduce *in vitro* the *in vivo* situation. The essential point here lies in the value of the beating frequency, which appears to be very similar between *in vivo* data from the literature and our ex vivo experiments.

Replacing the surrounding fluid with water may also induce difference in biochemical interactions. The mucociliary clearance process is generally described with three main components: mucins secreted by goblet that will give mucus, cilia that move the mucus and an ion transport process allowing maintaining an adequate aqueous environment on the airway epithelium [40]. These components can be, at least partially, controlled by local agonist, extracellular nucleotides, and nucleosides released from the epithelium. For example, it is known that ATP, UTP, and adenosine increase the ciliary beat frequency while ATP and UTP stimulate the secretion of mucins.

However, to our knowledge, the relation between these local agonists and the cilia beat pattern remains to be established. In 2000 Chilvers *et al*. proposed to use digital high speed video microscopy to visualize the beat pattern of human nasal cilia in the absence of mucus [19]. Since 2009, the European Respiratory Society strongly recommended to combine this technique (i.e., in the absence of mucus) with classical tests in order to ensure primary ciliary dyskinesia diagnosis [23, 41]. At this time, studying cilia beat pattern in the presence of mucus for patient specific evaluation would be very difficult to carry out because it would require tracheal or bronchial explants which are ethically problematic in clinical practice.

Clearly, the experimental procedure described in this study is not intended at evaluating the effect of possible alterations of the cilia environment (change in rheological properties of the mucus, modification of the nucleotides in the periciliary layer…). Our MBT experiments, the developed numerical model and the proposed index aim essentially at evaluating pathologies impairing mucociliary clearance resulting from a defect in ciliary motion (ciliopathies). In future studies, one could even envision adding exogenous drug to the survival medium to test the potential effect of a drug treatment. For now, the goal is to simulate the conditions of our experiment corresponding to a measurement that can be realized in a clinical setting.

### Conclusion

We have presented here a first experimental validation of the numerical model introduced in [25], describing the Newtonian fluid flow induced by cilia motion above a ciliated edge. This study shows that the computational model satisfactorily predicts the profile of velocities of micro-beads in the Newtonian flow, this profile being essentially parabolic. Recovering this profile allows us to assess the shear stress locally applied by the ciliated edge onto the fluid. Our model suggests that this shear stress at the cilia wall characterizes the momentum transfer between the cilia and the fluid, and thus the efficiency of the ciliary beating. Interestingly, the estimation of this index does not require any modification of the present clinical practice of data collection (nasal or bronchial brushing). This study opens the broad perspective of using this index in the future to characterize ciliary function of transport during normal and pathological conditions of either congenital origin such as PCD or acquired dyskinesia secondary to pathogenic invasion and/or occupational exposures.

## Supporting information

S1 Video Example n°1 of MBT movie. Patient №1, third edge presenting a good coordinated edge and good cila density.
(MP4)

S2 Video Example n°2 of MBT movie. Patient №4 presenting a good coordinated edge but relatively low cilia density.
(MP4)

S3 Video Example n°3 of MBT movie. Patient №10 presenting miscoordinated edge with non measurable metachronal wavelength.
(MP4)
